# β3 Integrin Promotes TGF-β1/H_2_O_2_/HOCl-Mediated Induction of Metastatic Phenotype of Hepatocellular Carcinoma Cells by Enhancing TGF-β1 Signaling

**DOI:** 10.1371/journal.pone.0079857

**Published:** 2013-11-18

**Authors:** Xin-Xia Feng, Mei Liu, Wei Yan, Zhen-Zhen Zhou, Yu-Jia Xia, Wei Tu, Pei-Yuan Li, De-An Tian

**Affiliations:** Department of Gastroenterology, Tongji Hospital, Tongji Medical College, Huazhong University of Science & Technology, Wuhan, The People’s Republic of China; Texas Tech University Health Sciences Center, United States of America

## Abstract

In addition to being an important mediator of migration and invasion of tumor cells, β3 integrin can also enhance TGF-β1 signaling. However, it is not known whether β3 might influence the induction of metastatic phenotype of tumor cells, especially non-metastatic tumor cells which express low level of β3. Here we report that H_2_O_2_ and HOCl, the reactive oxygen species produced by neutrophils, could cooperate with TGF-β1 to induce metastatic phenotype of non-metastatic hepatocellular carcinoma (HCC) cells. TGF-β1/H_2_O_2_/HOCl, but not TGF-β1 or H_2_O_2_/HOCl, induced β3 expression by triggering the enhanced activation of p38 MAPK. Intriguingly, β3 in turn promoted TGF-β1/H_2_O_2_/HOCl-mediated induction of metastatic phenotype of HCC cells by enhancing TGF-β1 signaling. β3 promoted TGF-β1/H_2_O_2_/HOCl-induced expression of itself via positive feed-back effect on p38 MAPK activation, and also promoted TGF-β1/H_2_O_2_/HOCl-induced expression of α3 and SNAI2 by enhancing the activation of ERK pathway, thus resulting in higher invasive capacity of HCC cells. By enhancing MAPK activation, β3 enabled TGF-β1 to augment the promoting effect of H_2_O_2_/HOCl on anoikis-resistance of HCC cells. TGF-β1/H_2_O_2_/HOCl-induced metastatic phenotype was sufficient for HCC cells to extravasate from circulation and form metastatic foci in an experimental metastasis model in nude mice. Inhibiting the function of β3 could suppress or abrogate the promoting effects of TGF-β1/H_2_O_2_/HOCl on invasive capacity, anoikis-resistance, and extravasation of HCC cells. These results suggest that β3 could function as a modulator to promote TGF-β1/H_2_O_2_/HOCl-mediated induction of metastatic phenotype of non-metastatic tumor cells, and that targeting β3 might be a potential approach in preventing the induction of metastatic phenotype of non-metastatic tumor cells.

## Introduction

Integrin expression is crucial for the migratory and invasive capability of tumor cells. Hepatocellular carcinoma (HCC) cells express several integrins which have been identified as the mediators of their migration and invasion, including α1β1, α2β1, α3β1, α6β1, αvβ1, αvβ3, and αvβ5 [1]–[6]. Most of these α and β integrin subunits are moderately expressed in non-metastatic HCC cells [3], [6], [7], whereas the expressions of α3 and β3 in these cells are very low or even negligible [4]–[9]. α3 and β3 are expressed in metastatic HCC cells [3]–[5], indicating that the up-regulation of α3 and β3 might be crucial for non-metastatic HCC cells to acquire metastatic phenotype. Moreover, β3 has also been found to modulate transforming growth factor β1 (TGF-β1) signaling in some types of cells [10], [11]. However, it is not known whether β3 might be involved in the induction of metastatic phenotype of tumor cells by functioning as modulatory factor.

Previous studies showed that TGF-β1 can induce α3 expression in non-metastatic HCC cells [1], [7], and suggested the idea that in hepatocellular carcinoma patients TGF-β1 triggers invasiveness of HCC cells by stimulating the expression of α3 integrin [1]. However, α3 expression is required but not sufficient for the invasiveness of HCC cells, since TGF-β1-treated non-metastatic HCC cells showed higher invasiveness only in the presence of exogenous matrix metalloproteinase (MMP) [1]. Given that αvβ3 could increase the invasive capacity of HCC cells [5], simultaneous up-regulation of both α3 and β3 might be required for higher invasiveness of HCC cells. Current knowledge of expression and function of β3 in non-metastatic HCC cells is very limited. TGF-β1 has been found to up-regulate β3 expression in other types of cells by activating p38 MAPK pathway, whilst β3 positively controls TGF-β1-induced p38 MAPK activation by promoting Src-mediated tyrosine phosphorylation of TβRII [10], [11]. However, TGF-β1 was inefficient in up-regulating β3 expression in non-metastatic HCC cells [9], implying that TGF-β1 might be less efficient in inducing p38 MAPK activation in these cells. In this context, other factors which could promote the activation of p38 MAPK might cooperate with TGF-β1 to up-regulate β3 expression in non-metastatic HCC cells.

The higher density of intratumoral neutrophils in hepatocellular carcinoma has been found to promote tumor metastasis [12], [13]. Neutrophil-derived H_2_O_2_ and HOCl, especially HOCl, could inhibit the activity of protein tyrosine phosphatases (PTPs) which negatively regulate the activation of MAPK pathways [14], [15]. Extracellular H_2_O_2_ could activate MAPK pathways [15]–[17]. Therefore, H_2_O_2_ and HOCl might be potential candidates for cooperating with TGF-β1 to induce the expression of β3 in HCC cells. In this study, we investigated whether H_2_O_2_ and HOCl could cooperate with TGF-β1 to induce the metastatic phenotype of non-metastatic HCC cells, and whether β3 expression is required for the induction. Our data showed that TGF-β1 could up-regulate the expression of β3 in presence of H_2_O_2_/HOCl. Intriguingly, β3 promoted TGF-β1/H_2_O_2_/HOCl-induced expression of α3 and SNAI2, and also enabled TGF-β1 to augment the promoting effect of H_2_O_2_/HOCl on anoikis-resistance, thus promoting TGF-β1/H_2_O_2_/HOCl-mediated induction of metastatic phenotype of HCC cells.

## Results

### H_2_O_2_/HOCl cooperates with TGF-β1 to induce higher invasive capacity of HCC cells

**Figure 1:**
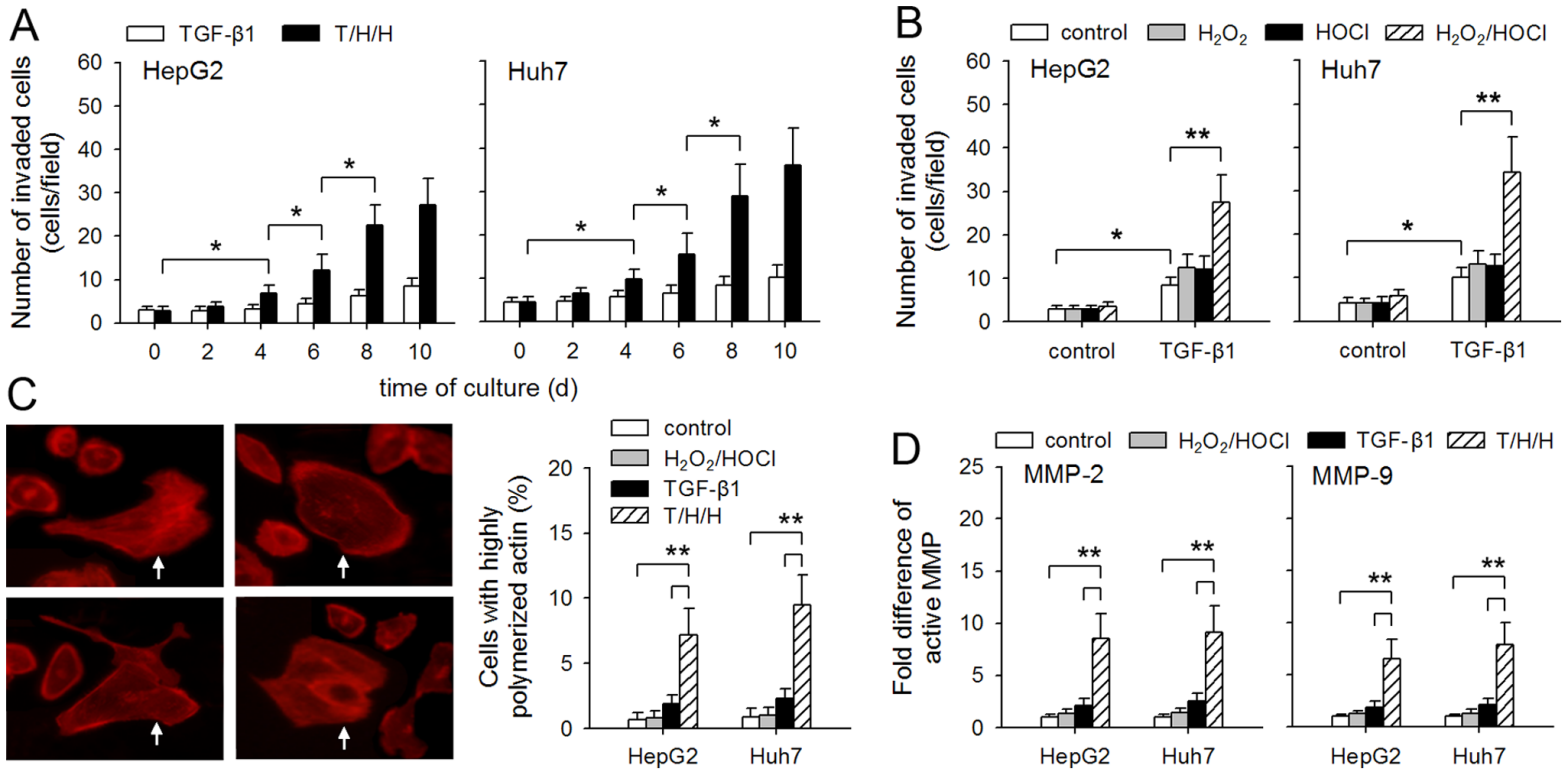
TGF-β1/H_2_O_2_/HOCl facilitates invasive capability of HCC cells. (**A**) Tumor cells were cultured in presence of TGF-β1 or T/H/H (TGF-β1, 5 ng/ml, H_2_O_2_, 100 µM, HOCl, 50 µM) for the indicated time, and then used for Matrigel invasion assay. (**B**) After 10-d culture in absence or presence of TGF-β1, H_2_O_2_ and HOCl, tumor cells were used for Matrigel invasion assay. (**C**) Tumor cells were treated for 10 days with H_2_O_2_/HOCl, TGF-β1, or T/H/H, and then incubated in presence of matrigel for 5 h. The cells with highly polymerized actin were visualized by staining with rhodamine-phalloidin (left). Their percentage in total cells was calculated (right). (**D**) Tumor cells were treated as described in C, and then cultured in presence of matrigel for 48 h. The MMP-2 and MMP-9 in supernatants were detected by zymography assay. The fold difference of active MMP-2 and MMP-9 was calculated after densitometric analysis of the gel. *P* values, *P < 0.05, **P < 0.01.

To investigate whether H_2_O_2_ and HOCl could cooperate with TGF-β1 to induce the metastatic phenotype of non-metastatic HCC cells, we first analyzed the effect of TGF-β1, H_2_O_2_ and HOCl on invasive capacity of HepG2 and Huh7 cells. The result showed that the invasive capacity of tumor cells was gradually increased after prolonged treatment (Figure 1A). Much higher invasive capacity of tumor cells was induced by TGF-β1 in presence of both H_2_O_2_ and HOCl, but not each of them alone (Figure 1B). Consistently, TGF-β1/H_2_O_2_/HOCl was most efficient in promoting the polymerization of actin in tumor cells (Figure 1C) and the production of active MMP-2 and MMP-9 by tumor cells (Figure 1D) in response to ECM molecules (matrigel), which are important for migratory and invasive properties of tumor cells [18]–[20]. These results indicated that TGF-β1 could induce much higher invasive capacity of HCC cells in presence of H_2_O_2_/HOCl, whereas TGF-β1 alone was less efficient.

### TGF-β1/H_2_O_2_/HOCl induces metastatic phenotype of HCC cells

**Figure 2:**
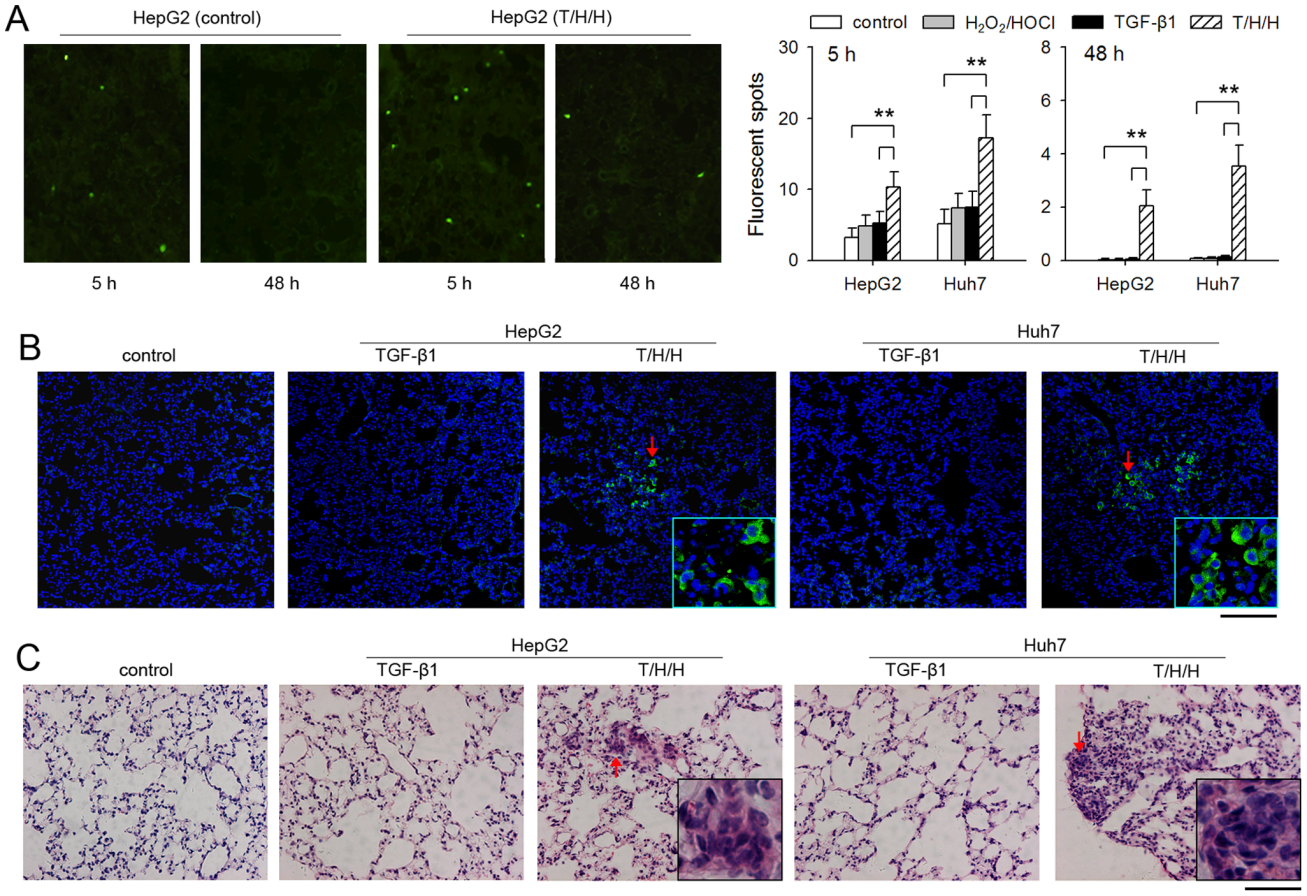
TGF-β1/H_2_O_2_/HOCl induces metastatic phenotype of tumor cells. (**A**) Tumor cells were cultured for 10 days in absence or presence of H_2_O_2_/HOCl, TGF-β1, and T/H/H (TGF-β1, 5 ng/ml, H_2_O­_2_, 100 µM, HOCl, 50 µM). The cells were then used for assay of tumor cell arrest and extravasation as described in Methods. The tumor cells in frozen sections were visualized by fluorescence microscopy (left). The average number of fluorescent spots per field was calculated (right). (**B and C**) Tumor cells were pre-treated for 10 days with TGF-β1 or T/H/H, and then injected into mice (n = 6 per group) via tail vein. Mice were sacrificed 4 weeks after inoculation. (**B**) Lung tissue sections were prepared and stained for HDGF (green) to identify the metastatic foci. Cell nuclei were stained with 4',6-diamidino-2-phenylindole (DAPI, blue). (**C**) Lung tissue sections were prepared and subjected to H&E staining. Representative photographs are shown. Bar, 100 µm. Insets are the high-power view of metastatic foci in corresponding picture indicated by arrow. **P<0.01.

We then tested the metastatic capability of HCC cells by using an experimental metastasis model in nude mice. Tumor cell arrest and extravasation in the lung of mice were assessed 5 h and 48 h, respectively, after i.v. injection of tumor cells. The pre-treatment with TGF-β1/H_2_O_2_/HOCl increased tumor cell arrest and resulted in the extravasation of tumor cells in the lung (Figure 2A), whereas pre-treatment with TGF-β1 or H_2_O_2_/HOCl did not promote tumor cell extravasation (Figure 2A). After inoculation via tail vein, the metastatic foci were only observed in the lung tissues of the mice inoculated with the tumor cells pre-treated with TGF-β1/H_2_O_2_/HOCl (Figure 2B, 2C). These results demonstrated that TGF-β1/H_2_O_2_/HOCl could induce the metastatic phenotype of HCC cells.

### H_2_O_2_/HOCl cooperates with TGF-β1 to up-regulate β3 expression

**Figure 3:**
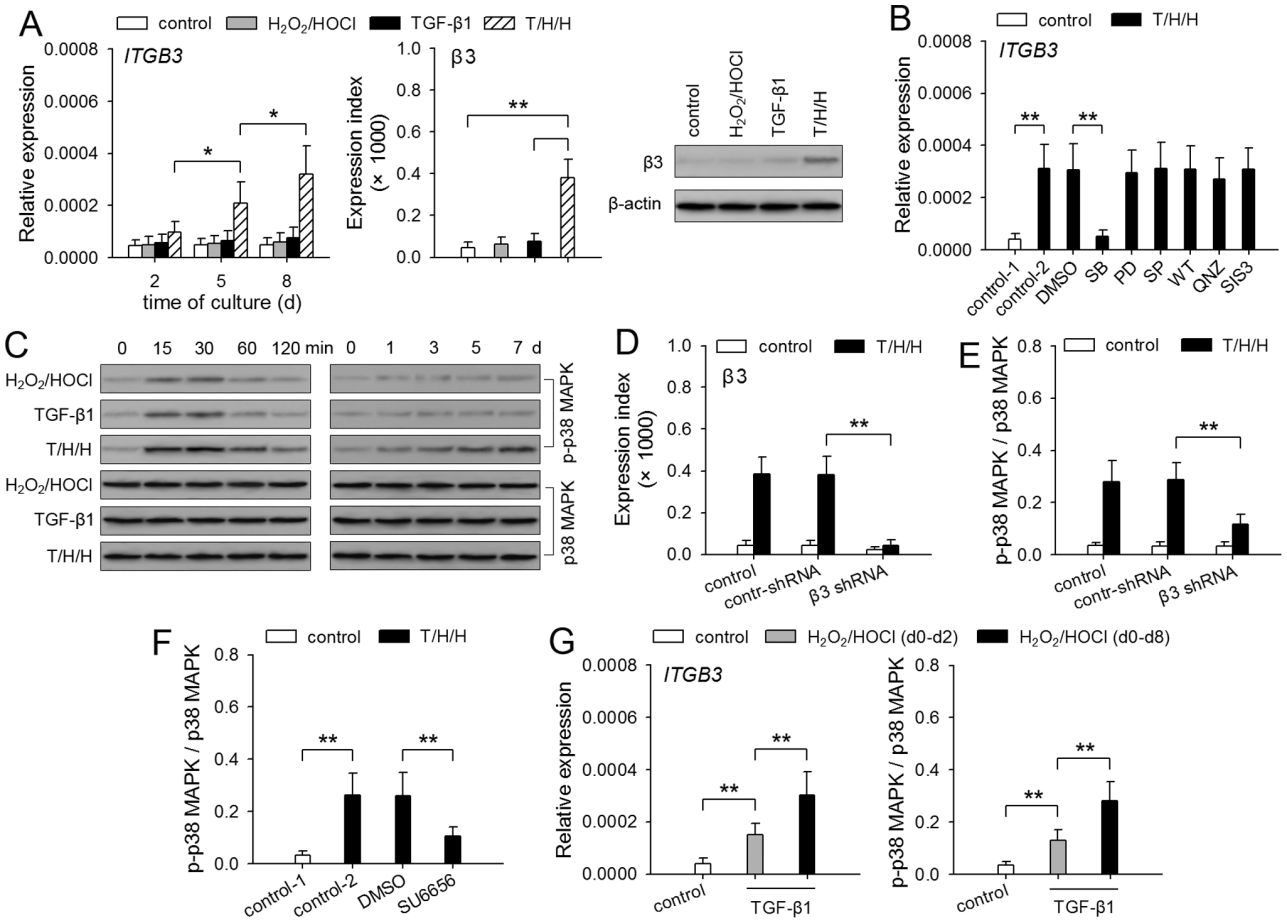
H_2_O_2_/HOCl cooperates with TGF-β1 to promote β3 expression and p38 MAPK activation. (**A**) HepG2 cells were stimulated with H_2_O_2_ (100 µM)/HOCl (50 µM), TGF-β1 (5 ng/ml), and T/H/H (TGF-β1/H_2_O_2_/HOCl). *ITGB3* expression was detected by real-time RT-PCR at the indicated time points, or by flow cytometry and Western blot after 8-d culture. (**B**) HepG2 cells were untreated or treated for 8 days with T/H/H in absence or presence of SB203580 (10 µM), PD98059 (10 µM), SP600125 (10 µM), wortmannin (WT, 40 nM), QNZ (40 nM), and SIS3 (2 µM). *ITGB3* expression was detected by real-time RT-PCR. (**C**) HepG2 cells were stimulated with H_2_O_2_/HOCl, TGF-β1, and T/H/H. The phosphorylation of p38 MAPK was detected by Western blot at the indicated time points. (**D and E**) Control HepG2 cells and the HepG2 cells expressing control shRNA or β3 shRNA were untreated or treated for 8 days with T/H/H. β3 expression was detected by flow cytometry (D). The relative activation of p38 MAPK (p-p38 MAPK/p38 MAPK) was calculated after densitometric analysis of Western blots (E). (**F**) HepG2 cells were untreated or treated for 8 days with T/H/H in absence or presence of SU6656 (10 µM). The relative activation of p38 MAPK was calculated after densitometric analysis of Western blots. (**G**) HepG2 cells were untreated or treated with TGF-β1 in presence of H_2_O_2_/HOCl within the indicated time-frame. *ITGB3* expression was detected by real-time RT-PCR. The relative activation of p38 MAPK was calculated after densitometric analysis of Western blots. *P* values, *P<0.05, **P<0.01.

We next focused on the effect of TGF-β1 and H_2_O_2_/HOCl on the expression of β3. TGF-β1 or H_2_O_2_/HOCl did not significantly influence the expression of β3 (Figure 3A). However, the expression of *ITGB3* gene was gradually increased after stimulation with TGF-β1/H_2_O_2_/HOCl (Figure 3A), indicating that H_2_O_2_/HOCl could cooperate with TGF-β1 to induce the expression of β3. We then stimulated HepG2 cells with TGF-β1/H_2_O_2_/HOCl in presence of SB203580 (p38 MAPK inhibitor), PD98059 (inhibitor of ERK pathway), SP600125 (JNK inhibitor), wortmannin (PI3K inhibitor), QNZ (NF-κB inhibitor), and SIS3 (Smad3 inhibitor). The inhibitory effect of each inhibitor on the corresponding signaling pathway was demonstrated by detecting the phosphorylation of down-stream target protein or the expression of target gene (Figure S1). The up-regulation of *ITGB3* expression was completely suppressed by p38 MAPK inhibitor, but not by other inhibitors (Figure 3B), indicating that p38 MAPK pathway was crucial for TGF-β1/H_2_O_2_/HOCl-induced up-regulation of β3 expression.

TGF-β1 was inefficient in inducing the sustained activation of p38 MAPK in HepG2 cells (Figure 3C). Co-stimulation with TGF-β1/H_2_O_2_/HOCl enhanced the transient activation of p38 MAPK, and also gradually enhanced the sustained activation of p38 MAPK (Figure 3C). To ascertain whether β3 was involved in the enhancement of the sustained activation of p38 MAPK, we used β3 shRNA to suppress the up-regulation of β3 expression (Figure 3D). Intriguingly, β3 shRNA significantly reduced the phosphorylation level of p38 MAPK induced by TGF-β1/H_2_O_2_/HOCl (Figure 3E), suggesting that β3 promoted TGF-β1-induced activation of p38 MAPK pathway. To confirm this, we stimulated HepG2 cells with TGF-β1/H_2_O_2_/HOCl in presence of SU6656 (Src inhibitor), since inhibiting Src activity could prevent the ability of β3 integrin to enhance TGF-β1 signaling [10], [11]. The result showed that SU6656 significantly reduced the phosphorylation level of p38 MAPK induced by TGF-β1/H_2_O_2_/HOCl (Figure 3F, S1), suggesting that β3 augmented p38 MAPK activation by enhancing TGF-β1 signaling.

To further clarify the role of H_2_O_2_/HOCl, we removed H_2_O_2_/HOCl 48 h after stimulation with TGF-β1/H_2_O_2_/HOCl, and continuously stimulated HepG2 cells with TGF-β1. The result showed that both *ITGB3* expression and the phosphorylation level of p38 MAPK were significantly reduced if H_2_O_2_ and HOCl were removed (Figure 3G), suggesting that the continuous existence of H_2_O_2_/HOCl was required for inducing higher activation level of p38 MAPK and higher expression of *ITGB3* gene.

### β3 augments the promoting effect of TGF-β1/H_2_O_2_/HOCl on invasive capacity

**Figure 4:**
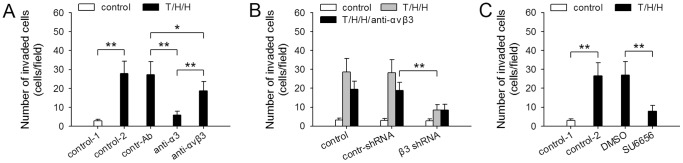
β3 augments the promoting effect of TGF-β1/H_2_O_2_/HOCl on invasive capacity. (**A**) HepG2 cells were untreated or treated with T/H/H (TGF-β1/H_2_O_2_/HOCl) for 10 days, and then used for Matrigel invasion assay in absence or presence of control antibody, anti-α3 antibody, anti-αvβ3 antibody. (**B**) Control HepG2 cells and the HepG2 cells expressing control shRNA or β3 shRNA were untreated or treated for 10 days with T/H/H, and then used for Matrigel invasion assay in absence or presence of anti-αvβ3 antibody. (**C**) HepG2 cells were untreated or treated for 10 days with T/H/H in absence or presence of SU6656 (10 µM). The cells were then used for Matrigel invasion assay. *P* values, *P<0.05, **P<0.01.

To ascertain the role of β3 integrin in TGF-β1/H_2_O_2_/HOCl-induced invasiveness, we further detected the invasive migration of TGF-β1/H_2_O_2_/HOCl-treated HepG2 cells in presence of α3 and αvβ3 blocking antibodies. Blocking α3 almost abolished the invasiveness of HepG2 cells. Blocking αvβ3 partially but significantly suppressed the invasive migration (Figure 4A). Intriguingly, if up-regulation of β3 expression in HepG2 cells was suppressed by shRNA, TGF-β1/H_2_O_2_/HOCl induced much lower invasive capacity of the cells (Figure 4B). Moreover, inhibiting Src activity with SU6656 significantly suppressed the promoting effect of TGF-β1/H_2_O_2_/HOCl on invasive capacity of HepG2 cells (Figure 4C). On the other hand, HepG2 cells did not acquire higher invasive capacity when β3 was overexpressed in the cells only by transfection with β3 expression vector (data not shown), indicating that β3 alone could not increase the invasive capacity of non-metastatic HCC cells without the stimulation with TGF-β1/H_2_O_2_/HOCl. These results suggested that in addition to being a mediator of invasive migration, β3 integrin could function as a modulator to promote the effect of TGF-β1/H_2_O_2_/HOCl on invasiveness of HCC cells by enhancing TGF-β1 signaling.

### β3 promotes TGF-β1/H_2_O_2_/HOCl-mediated up-regulation of α3 and SNAI2 expression

**Figure 5:**
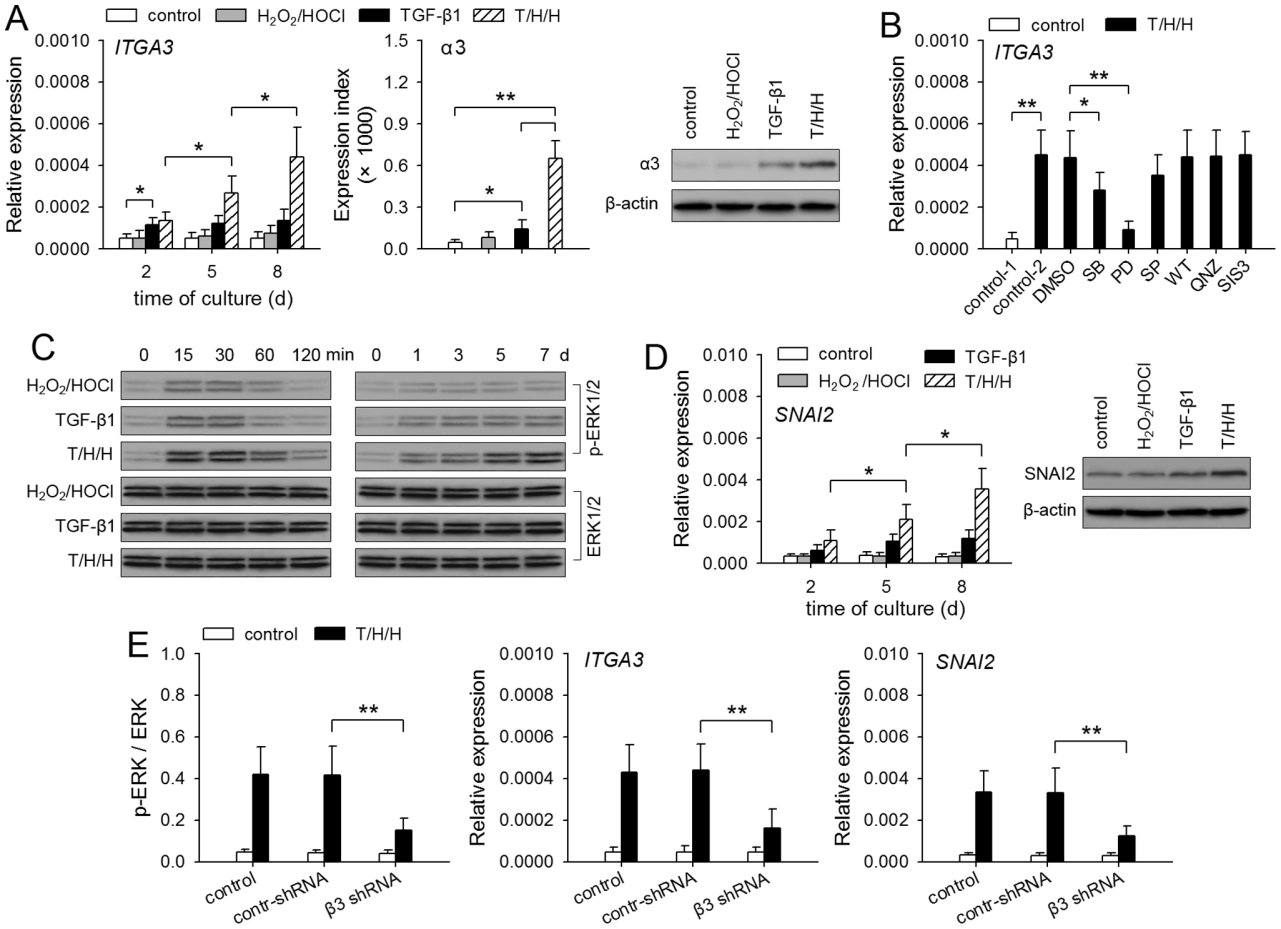
β3 promotes TGF-β1/H_2_O_2_/HOCl-induced expression of α3 and SNAI2. (**A**) HepG2 cells were stimulated with H_2_O_2_/HOCl, TGF-β1, and T/H/H (TGF-β1/H_2_O­_2_/HOCl). *ITGA3* expression was detected by real-time RT-PCR at the indicated time points, or by flow cytometry and Western blot after 8-d culture. (**B**) HepG2 cells were untreated or treated for 8 days with T/H/H in absence or presence of SB203580 (10 µM), PD98059 (10 µM), SP600125 (10 µM), wortmannin (WT, 40 nM), QNZ (40 nM), and SIS3 (2 µM). *ITGA3* expression was detected by real-time RT-PCR. (**C and D**) HepG2 cells were stimulated with H_2_O_2_/HOCl, TGF-β1, and T/H/H. Phosphorylated ERK was detected by Western blot at the indicated time points (C). *SNAI2* expression was detected by real-time RT-PCR at the indicated time points, or by Western blot after 8-d culture (D). (**E**) Control HepG2 cells and the HepG2 cells expressing control shRNA or β3 shRNA were untreated or treated for 8 days with T/H/H. The relative activation of ERK (p-ERK/ERK) was calculated after densitometric analysis of Western blots. The expression of *ITGA3* and *SNAI2* was detected by real-time RT-PCR. *P* values, *P<0.05, **P<0.01.

Based on the above results, we next investigated whether β3 might influence the expression of α3. TGF-β1 induced α3 expression in HepG2 cells (Figure 5A). Intriguingly, much higher expression level of α3 was induced by TGF-β1/H_2_O_2_/HOCl. We then analyzed the signaling pathway(s) involved in up-regulation of *ITGA3* expression by stimulating HepG2 cells with TGF-β1/H_2_O_2_/HOCl in presence of SB203580, PD98059, SP600125, wortmannin, QNZ, and SIS3. The result showed that p38 MAPK, ERK, and JNK pathways were involved in up-regulating *ITGA3* expression (Figure 5B). Among them, ERK pathway was the most efficient one. In line with this, the sustained activation of ERK pathway in HepG2 cells was gradually enhanced by stimulation with TGF-β1/H_2_O_2_/HOCl (Figure 5C).

Both Smad and ERK pathways are involved in up-regulating the expression of SNAI2 [21] which positively controls α3β1-mediated migration of tumor cells [22]. TGF-β1-induced activation of Smad pathway was also gradually enhanced in the presence of H_2_O_2_/HOCl (Figure S2). Consistently, TGF-β1/H_2_O_2_/HOCl induced higher expression of SNAI2 in HepG2 cells (Figure 5D). Inhibiting β3 expression with shRNA did not influence the activation of Smad pathway (data not shown), but suppressed TGF-β1/H_2_O_2_/HOCl-induced activation of ERK, and also suppressed the up-regulation of *ITGA3* and *SNAI2* expression (Figure 5E). Taken together, these results suggested that the up-regulation of β3 enhanced the sustained activation of ERK pathway, thus promoting TGF-β1/H_2_O_2_/HOCl-induced expression of both α3 and SNAI2.

The above results suggested that the higher and sustained activation of p38 MAPK, ERK, and Smad pathways was necessary for TGF-β1/H_2_O_2_/HOCl to induce the invasive capacity of HCC cells. To further confirm this, we added SB203580, PD98059, and SIS3 to the cell culture 96 h after stimulation and thereafter. Each of these inhibitors significantly suppressed the promoting effect of TGF-β1/H_2_O_2_/HOCl on invasive migration and extravasation of HepG2 cells (Figure S3), suggesting that the sustained activation of these pathways was indeed required for TGF-β1/H_2_O_2_/HOCl to induce higher invasive capacity of HCC cells.

### β3 enables TGF-β1 to promote the anoikis-resistance of HCC cells

TGF-β1 has the potential to induce apoptosis of tumor cells in a Smad-dependent manner [23]. We therefore further investigated whether TGF-β1/H_2_O_2_/HOCl might increase or decrease the apoptosis-resistance of HCC cells. TGF-β1 could induce transient activation of Smad pathway, but was inefficient in inducing the sustained activation of Smad pathway in HCC cells (Figure S2). Consistently, the treatment with TGF-β1 alone promoted the apoptosis of HepG2 cells after 48-h culture, whereas the apoptosis was gradually reduced after prolonged stimulation (Figure S4A). Importantly, the apoptosis of HepG2 cells was further reduced in presence of H_2_O_2_/HOCl (Figure S4A).

**Figure 6:**
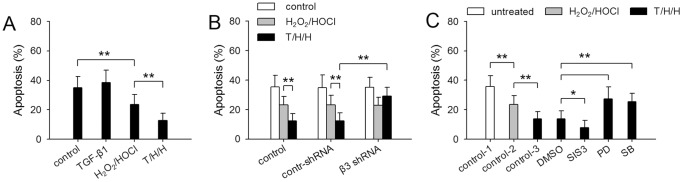
β3 enables TGF-β1 to promote anoikis-resistance of HCC cells. (**A**) HepG2 cells were untreated or treated for 10 days with TGF-β1, H_2_O­_2_/HOCl, or T/H/H (TGF-β1/H_2_O_2_/HOCl). The cells were then used for the assay of anoikis as described in Methods. (**B**) Control HepG2 cells and the HepG2 cells expressing control shRNA or β3 shRNA were untreated or treated for 10 days with H_2_O­_2_/HOCl or T/H/H. The cells were then used for the assay of anoikis. (**C**) HepG2 cells were treated with T/H/H for 10 days in absence or presence of SB203580 (10 µM), PD98059 (10 µM), and SIS3 (2 µM), and then used for the assay of anoikis. Untreated and H_2_O­_2_/HOCl-treated cells were used as control. *P* values, *P<0.05, **P<0.01.

The prolonged treatment with TGF-β1/H_2_O_2_/HOCl reduced the expression of pro-apoptotic genes (*BAX*, *BIM*, *BID*), and increased the expression of anti-apoptotic genes (*MCL1*, *BCL2*, *c-FLIP*) (Figure S4B). These genes also influence mitochondrial pathway and extrinsic pathway involved in anoikis [24], [25]. We therefore further investigated whether the treatment with TGF-β1/H_2_O_2_/HOCl might increase the anoikis-resistance of HepG2 cells. Pre-treatment with TGF-β1 alone slightly increased the apoptosis of tumor cells cultured under anchorage-independent condition (anoikis) (Figure 6A). The anoikis of tumor cells was reduced by the pre-treatment with H_2_O_2_/HOCl. Intriguingly, TGF-β1 augmented the effect of H_2_O_2_/HOCl (Figure 6A). However, if β3 expression was suppressed with shRNA, TGF-β1 could not augment the promoting effect of H_2_O_2_/HOCl on anoikis-resistance (Figure 6B). We therefore further analyzed the effect of Smad and MAPK pathways on anoikis-resistance. The results showed that inhibiting Smad3 further reduced anoikis of HCC cells, whereas inhibiting MAPK pathways increased the anoikis of the cells (Figure 6C). These results suggested that the up-regulation of β3 enabled TGF-β1 to promote anoikis-resistance by enhancing the activation of MAPK pathways.

### β3 is required for TGF-β1/H_2_O_2_/HOCl-mediated induction of metastatic phenotype

**Figure 7:**
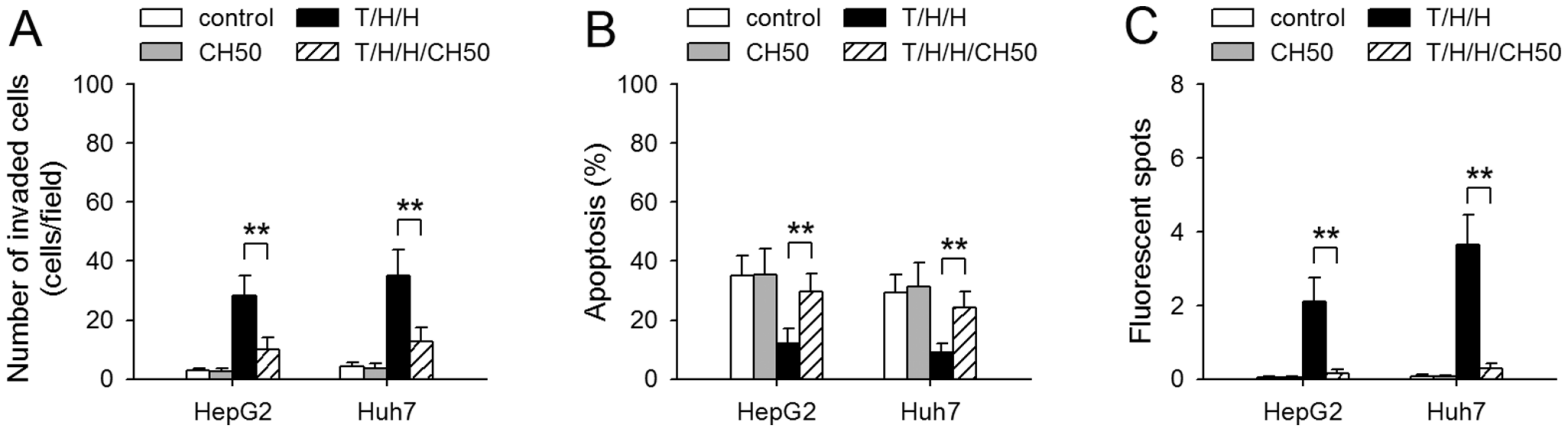
CH50 suppresses the promoting effect of TGF-β1/H_2_O_2_/HOCl on metastatic capability of HCC cells. HepG2 and Huh7 cells were untreated or treated for 10 days with T/H/H (TGF-β1/H_2_O_2_/HOCl) in absence or presence of CH50 (20 µg/ml). The cells were then used for the assay of invasive migration (**A**), anoikis (**B**), and extravasation (**C**) as described in Methods. *P* values, **P<0.01.

To further confirm the requirement of β3 for TGF-β1/H_2_O_2_/HOCl-mediated induction of metastatic phenotype, we treated HCC cells with TGF-β1/H_2_O_2_/HOCl in presence of CH50, a recombinant polypeptide which suppresses the function of αvβ3 [26]. CH50 attenuated TGF-β1/H_2_O_2_/HOCl-induced activation of p38 MAPK and ERK pathways, but did not influence the activation of Smad pathway (Figure S5A). TGF-β1/H_2_O_2_/HOCl-induced expression of *ITGB3*, *ITGA3*, and *SNAI2* genes was suppressed by CH50 (Figure S5B). In presence of CH50, TGF-β1/H_2_O_2_/HOCl was inefficient in inducing invasive capacity (Figure 7A), anoikis-resistance (Figure 7B), and extravasation of HCC cells (Figure 7C). These results suggested that the function of β3 was indeed important for TGF-β1/H_2_O_2_/HOCl-mediated induction of metastatic phenotype of non-metastatic HCC cells.

## Discussion

Both extrahepatic metastasis and intrahepatic metastasis of HCC cells involve the step of extravasation from circulation [27], [28], which requires higher invasive capacity and anoikis-resistance of tumor cells. TGF-β1 could induce the invasive capacity of non-metastatic HCC cells to some extent as shown by our data and others [1], [7]. Nevertheless, TGF-β1-treated HCC cells were unable to extravasate from circulation. Our data in present study showed that H_2_O_2_/HOCl could cooperate with TGF-β1 to induce higher invasive capacity and anoikis-resistance of non-metastatic HCC cells. Consistently, TGF-β1/H_2_O_2_/HOCl-induced metastatic phenotype was sufficient for HCC cells to extravasate from circulation and form metastatic foci in the secondary sites. H_2_O_2_/HOCl enhanced TGF-β1 signaling, which was crucial for inducing higher invasive capacity and anoikis-resistance of non-metastatic HCC cells. Importantly, β3 played an indispensable role in enhancing TGF-β1 signaling, and therefore was required for TGF-β1/H_2_O_2_/HOCl-mediated induction of metastatic phenotype of non-metastatic HCC cells.

The prolonged treatment with TGF-β1/H_2_O_2_/HOCl was required for inducing the metastatic phenotype of non-metastatic HCC cells, since the expression of β3 was gradually increased. Our data showed that TGF-β1 was inefficient in inducing the expression of β3 in non-metastatic HCC cells, which is consistent with the result reported by Nejjari *et al* [9]. The activation of p38 MAPK pathway induced by either TGF-β1 or H_2_O_2_/HOCl was not sufficient for up-regulating β3 expression, suggesting that the sustained and higher activation of p38 MAPK pathway was required for inducing β3 expression in non-metastatic HCC cells. H_2_O_2_/HOCl cooperated with TGF-β1 to induce higher activation level of p38 MAPK, thus up-regulating the expression of β3. The requirement for the continuous existence of H_2_O_2_/HOCl implicated that the attenuation of PTP activity was required for the sustained activation of p38 MAPK pathway. On the other hand, the up-regulation of β3 in turn enhanced TGF-β1 signaling, resulting in the gradually enhanced activation of p38 MAPK pathway in non-metastatic HCC cells. If the up-regulation of β3 expression was suppressed, the sustained activation of p38 MAPK was maintained at much lower level. Moreover, if the function of β3 was suppressed, TGF-β1/H_2_O_2_/HOCl-induced activation of p38 MAPK was not sufficient for inducing higher expression of β3. Therefore, H_2_O_2_/HOCl cooperation with TGF-β1 actually augmented p38 MAPK-β3 feed-back regulation, thus resulting in the gradual increase of both β3 expression and p38 MAPK activation. Since the expression of β3 was very low in non-metastatic HCC cells, the feed-back regulation was gradually enhanced, which might explain the requirement for the prolonged stimulation with TGF-β1/H_2_O_2_/HOCl.

In presence of H_2_O_2_/HOCl, TGF-β1-induced activation of Smad and MAPK pathways was gradually enhanced. H_2_O_2_/HOCl promoted the sustained activation of Smad pathway by down-regulating the expression of Nm23-H1 (our unpublished data), whereas the up-regulation of β3 expression was crucial for the enhanced activation of MAPK pathways. Inhibiting the expression and function of β3 did not influence the activation of Smad pathway in HCC cells, suggesting that β3 could not influence the activity of TβRI. It has been found that β3 regulates TGF-β signaling by interacting physically with TβRII and promoting Src-mediated tyrosine phosphorylation of TβRII, which is essential for the ability of TGF-β1 to activate MAPKs [10], [11]. Our data showed that inhibiting either β3 or Src could significantly suppress the sustained activation of MAPK pathways after prolonged stimulation with TGF-β1/H_2_O_2_/HOCl, suggesting that β3-Src-mediated modulation of TβRII was crucial for the higher and sustained activation of MAPK pathways. Importantly, inducing higher and sustained activation of MAPK pathways was necessary for TGF-β1/H_2_O_2_/HOCl to induce higher invasive capacity and anoikis-resistance of non-metastatic HCC cells.

The up-regulation of β3 resulted in the higher expression of both α3 and SNAI2 by enhancing the activation of MAPK pathways. Previous study showed that TGF-β1 induced α3 expression in non-metastatic HCC cells, but the cells did not secrete MMP [1]. The reason might be that TGF-β1 alone could not induce higher expression of SNAI2 in non-metastatic HCC cells as shown by our data. SNAI2 has a positive effect on α3β1-mediated invasiveness of tumor cells [22], since SNAI2 promotes the production of MMP-2 and MMP-9 [19], [20]. Both Smad and ERK pathways are involved in up-regulating the expression of SNAI2 [21]. TGF-β1/H_2_O_2_/HOCl, but not TGF-β1 alone, induced much higher expression of SNAI2 by inducing higher and sustained activation of both Smad and ERK pathway. Although β3 did not influence the activation of Smad pathway, its enhancing effect on the activation of ERK pathway was indispensable for the up-regulation of SNAI2 expression. Inhibiting the expression or function of β3 could significantly suppress the expression of SNAI2. Our result is also supported by another report that inhibiting ERK signaling blocked TGF-β1-induced SNAI2 expression in oral squamous cell carcinoma cells [20]. Therefore, up-regulation of β3 was crucial for TGF-β1/H_2_O_2_/HOCl to induce higher expression of SNAI2 in non-metastatic HCC cells. On the other hand, TGF-β1/H_2_O_2_/HOCl could induce much higher expression of α3 due to positive effect of β3 on the sustained activation of p38 MAPK and ERK pathways. In this context, up-regulation of β3 could promote both α3 expression and α3β1-mediated invasive migration of HCC cells.

TGF-β1 has the potential to induce apoptosis of tumor cells in a Smad-dependent manner [23]. The treatment with TGF-β1 alone within a relatively short period of time could promote the apoptosis in HCC cells as shown by our data and others [29], whereas the apoptosis was reduced after prolonged stimulation, possibly due to the inefficiency of TGF-β1 in inducing the sustained activation of Smad pathway and the proliferation of surviving cells. TGF-β1 alone could not promote the anoikis-resistance of HCC cells, which might be one of the reasons that TGF-β1-treated HCC cells were unable to extravasate from circulation. H_2_O_2_/HOCl promoted the anoikis-resistance of HCC cells, since H_2_O_2_ and HOCl could activate NF-κB [30], [31], which can activate the expression of a group of antiapoptotic genes [32], [33]. Although the enhancement of TGF-β1-induced Smad activation by H_2_O_2_/HOCl might have negative effect on anoikis-resistance, the up-regulation of β3 reduced the effect of Smad pathway by enhancing the activation of MAPK pathways. The enhanced activation of MAPK pathways could promote apoptosis-resistance of HCC cells, and antagonize the negative effect of Smad pathway on apoptosis-resistance [23]. Therefore, the up-regulation of β3 enabled TGF-β1 to augment the promoting effect of H_2_O_2_/HOCl on anoikis-resistance. In line with this, TGF-β1 augmented the effect of H_2_O_2_/HOCl if β3 expression was up-regulated, but attenuated the effect of H_2_O_2_/HOCl if the up-regulation of β3 expression was suppressed.

In summary, in this study we demonstrated that β3 expression in non-metastatic HCC cells was up-regulated by TGF-β1 in presence of H_2_O_2_/HOCl. Importantly, β3 could promote TGF-β1/H_2_O_2_/HOCl-mediated induction of metastatic phenotype of non-metastatic tumor cells by enhancing TGF-β1 signaling. Simply increasing β3 expression might not be sufficient for promoting the metastatic capability, since β3 could not influence the activation of Smad pathway. However, TGF-β1/H_2_O_2_/HOCl could not induce the metastatic phenotype of HCC cells without β3. Our findings in this study suggest that targeting β3 might be a potential approach in preventing the induction of metastatic phenotype of non-metastatic tumor cells.

## Materials and Methods

### Ethics statement

All animal works were conducted according to relevant national and international guidelines. They were approved by the Committee on the Ethics of Animal Experiments of Tongji Medical College (Permit Number: 2011-S275) and monitored by the Department of Experimental Animals of Tongji Medical College.

### Cells and reagents

Human HCC cell lines HepG2 and Huh7 were purchased from China Center for Type Culture Collection (CCTCC, Wuhan, China) and cultured according to their guidelines. H_2_O_2_ and HOCl were purchased from Sigma-Aldrich (St. Louis, MO). TGF-β1 was purchased from PeproTech (Rocky Hill, NJ). SB203580, PD98059, SP600125, wortmannin, 6-amino-4-(4-phenoxyphenylethylamino) quinazoline (QNZ), SIS3, and SU6656 were purchased from Merck4Biosciences (Calbiochem). Recombinant polypeptide CH50 was prepared as described previously [34].

### Matrigel invasion assay

Matrigel invasion assay was performed using Boyden chambers (Transwell, Corning, Inc., Corning, NY). The transwell filters were coated with matrigel (BD Biosciences). The lower chambers were filled with DMEM medium containing 10% FBS. 1×10^5^ tumor cells were placed in the upper compartment. After 24-h incubation at 37°C in a humidified incubator with 5% CO_2_, the non-invading cells were removed. The invasive cells attached to the lower surface of membrane insert were fixed, stained, and counted under a microscope from 5 randomly chosen fields in each membrane. The average number of the cells per field was calculated. When indicated, the cells were pre-incubated with 10 µg/ml of anti-α3 antibody (Santa Cruz Biotechnology) or anti-αvβ3 antibody (Chemicon) for 30 min. Matrigel invasion assay was then performed in the presence of antibody.

### Analysis for actin polymerization

Tumor cells were incubated in matrigel-coated plate for 5 h. The cells were then fixed in 4% paraformaldehyde, permeabilized with 0.1% Triton X-100, and then stained with rhodamine-phalloidin (Invitrogen) according to the manufacturer’s protocol to visualize the cells with highly polymerized actin.

### MMP assay by gelatin zymography

Tumor cells were cultured for 48 h in DMEM medium containing 1% FBS in presence of pre-coated matrigel. The assay of MMP-2 and MMP-9 in supernatants was performed as described previously [35].

### Assay of tumor cell arrest and extravasation in lung

Athymic nude (nu/nu) mice (4–5 weeks old) were purchased from Beijing HFK Bio-Technology Co. LTD. (Beijing, China). The mice were maintained in the accredited animal facility of Tongji Medical College. Tumor cells were labeled with CFSE, and injected into mice via tail vein (2×10^6^ cells/mouse). Lungs of mice were harvested 5 h and 48 h after the injection. Frozen sections were prepared and analyzed by fluorescence microscopy. Fluorescent spots were counted from 20 randomly chosen fields in sections of each mouse.

### Immunofluorescence and histology

Tumor cells were injected into mice via tail vein (2×10^6^ cells/mouse). The lung tissues were harvested 4 weeks after inoculation. Frozen tissue sections were prepared and subjected to immunofluorescence analysis as previously described [36]. Anti-human HDGF (hepatoma-derived growth factor) antibody (Santa Cruz Biotechnology) was used as primary antibody. FITC-conjugated goat anti-rabbit IgG was used as secondary antibody. Images were obtained using a laser scanning confocal microscope (Olympus, FV500, Japan). For H&E staining, the lung tissues were embedded in paraffin according to standard histological procedures. Sections were stained with hematoxylin and eosin.

### Assay of gene expression by real-time RT-PCR

Total RNA was extracted from cells with TRIzol reagent (Invitrogen). The relative quantity of mRNA was determined by real-time RT-PCR according to MIQE guidelines [37]. *GAPDH*, *PPIA*, and *HPRT1* were chosen as reference genes. The relative expression of gene was calculated using GeNorm software. The primer sequences were as follows: *ITGB3*, sense 5'-CATCCTGGTGGTCCTGCTCT-3', antisense 5'-GCCTCTTTATACAGTGGGTTGTT-3'; *ITGA3*, sense 5'-ATACACTCCAGACCTCGCT-3', antisense 5'-GGCTTCCTACATCCTCC A-3'; *SNAI2*, sense 5′-AGGAATCTGGCTGCTGTG-3′, antisense 5′-GGAGAAATGCCT TTGGAC-3′; *BAX*, sense 5'-TTTTGCTTCAGGGTTTCAT C-3', antisense 5'-GACACTCGC TCAGCTTCTTG-3'; *BIM*, sense 5'-CAGAGCCACAAGACAGGA-3', antisense 5'-CCAT ACAAATCTAAGCCAGT-3'; *BID*, sense 5'-GCCGTCCTTGCTCCGTGAT-3', antisense 5'-ATGCCAGGGCTCCGTCTA-3'; *MCL1*, sense 5'-TTGACTTCTGTTTGTCTTACGCT-3', antisense 5'-TGGTCCTAACCCTTCCTGG-3'; *BCL2*, sense 5'-GGTCATGTGTGTGGAGA GC-3', antisense 5'-GATCCAGGTGTGCAGGTG-3'; *c-FLIP*, sense 5'-AGAGTGAGGCGAT TTGACCTG-3', antisense 5'-AAGGTGAGGGTTCCTGAGCA-3'. *GAPDH*, sense 5'-TCA TTGACTCAACTACATGGTTT-3', antisense 5'-GAAGATGGTGATGGGATTTC-3'; *PPIA*, sense 5'-GTCAACCCCACCGTGTTCTT-3', antisense 5'-CTGCTGTCTTTGGGACCTTG T-3'; *HPRT1*, sense 5'-GCTGAGGATTTGGAAAGGGTG-3', antisense 5'-CAGAGGGCTA CAATGTGATGG-3'.

### Flow cytometric analysis

Tumor cells were stained with FITC-conjugated mouse-anti-human β3 and α3 (Santa Cruz Biotechnology), or isotype control. Parameters were acquired on a FACS Calibur flow cytometer (BD Biosciences) and analyzed with CellQuest software (BD Biosciences). Percent staining was defined as the percentage of cells in the gate (M1) which was set to exclude ∼99% of isotype control cells. The expression index was calculated by using the formula: mean fluorescence × percentage of positively stained cells [38].

### Western blot assay

Western blot assay was done as described previously [39]. Abs were purchased from Santa Cruz Biotechnology (Santa Cruz, CA) and Cell Signaling Technology (Beverly, MA).

### Cell transfection

To suppress β3 expression, tumor cells were transduced with β3 shRNA(h) lentiviral particles, or control shRNA lentiviral particles (Santa Cruz Biotech, Inc.) according to the manufacturer’s protocol. After selection with puromycin, the cells were used for further experiments.

### Assay of apoptosis and anoikis

For the assay of apoptosis, tumor cells were cultured under the indicated conditions for the indicated time. For the assay of anoikis, tumor cells were cultured (1×10^6^/well) for 24 h in 6-well plates pre-coated with poly-HEMA (10 mg/ml, Sigma). The cells were then stained with Annexin V-FITC/Propidium Iodide (PI) apoptosis detection kit (BD Biosciences, San Diego, CA), and analyzed by flow cytometry.

### Statistics

Data are pooled from three independent experiments with a total of six samples in each group. Results were expressed as mean value ± SD and interpreted by one-way ANOVA. Differences were considered to be statistically significant when *P* < 0.05.

## Supporting Information

Figure S1 **The inhibitory effect of inhibitors on signaling pathways.** HepG2 cells were untreated or treated for 7 days with T/H/H in absence or presence of SB203580 (10 µM), PD98059 (10 µM), SP600125 (10 µM), wortmannin (WT, 40 nM), QNZ (40 nM), SIS3 (2 µM), and SU6656 (10 µM). The phosphorylation of MK2 was detected to demonstrate the inhibition of p38 MAPK by SB203580. The phosphorylation of ERK was detected to demonstrate the inhibition of MEK by PD98059. The phosphorylation of c-Jun was detected to demonstrate the inhibition of JNK by SP600125. The phosphorylation of Akt was detected to demonstrate the inhibition of PI3K by wortmannin. The expression of iASPP was detected to demonstrate the inhibition of NF-κB by QNZ. The expression of PAI-1 was detected to demonstrate the inhibition of Smad3 by SIS3. The phosphorylation of p38 MAPK was detected to demonstrate the inhibition of Src by SU6566.
(TIF)

Figure S2 **H_2_O_2_/HOCl promotes TGF-β1-induced sustained activation of Smad pathway.** HepG2 cells were stimulated with H_2_O_2_/HOCl, TGF-β1, and T/H/H (TGF-β1, 5 ng/ml, H_2_O­_2_, 100 µM, HOCl, 50 µM). The phosphorylation of Smad2 and Smad3 was detected by Western blot at the indicated time points.
(TIF)

Figure S3 **Sustained activation of signaling pathways is required for TGF-β1/H_2_O_2_/HOCl to promote invasion.** HepG2 cells were cultured in absence or presence of T/H/H (TGF-β1/H_2_O_2_/HOCl). After 96-h culture, SB203580 (20 µM), PD98059 (20 µM), or SIS3 (2 µM) was added to the culture containing TGF-β1/H_2_O­_2_/HOCl. The cells were continuously cultured for another 6 days, and then used for the assay of invasive migration (**A**) and extravasation (**B**) as described in Methods. *P* values, *P<0.05, **P<0.01.
(TIF)

Figure S4 **The effect of TGF-β1/H_2_O_2_/HOCl on apoptosis of HCC cells.** (**A**) HepG2 cells were cultured in absence or presence of H_2_O_2_/HOCl, TGF-β1, and T/H/H (TGF-β1/H_2_O­_2_/HOCl). The apoptosis of the cells was detected at the indicated time points as described in Methods. (**B**) HepG2 cells were cultured for 10 days in absence or presence of H_2_O_2_/HOCl, TGF-β1, or T/H/H. The expression of *BAX*, *BIM*, *BID*, *MCL1*, *BCL2*, and *c-FLIP* was detected by real-time RT-PCR and Western blot. *P* values, *P<0.05, **P<0.01.
(TIF)

Figure S5 **CH50 alters the effect of TGF-β1/H_2_O_2_/HOCl on HCC cells.** (**A**) HepG2 cells were untreated or treated for the indicated time with T/H/H (TGF-β1/H_2_O_2_/HOCl) in absence or presence of CH50 (20 µg/ml). The relative activation of p38 MAPK (p-p38 MAPK/p38 MAPK), ERK (p-ERK/ERK), Smad2 (p-Smad2/Smad2), and Smad3 (p-Smad3/Smad3) was calculated after densitometric analysis of Western blots. (**B**) HepG2 cells were untreated or treated for 8 days with T/H/H in absence or presence of CH50. The expression of *ITGB3*, *ITGA3*, and *SNAI2* genes was detected by real-time RT-PCR. *P* values, **P<0.01.
(TIF)
