# Supplementary figures and images for: β3 Integrin Promotes TGF-β1/H2O2/HOCl-Mediated Induction of Metastatic Phenotype of Hepatocellular Carcinoma Cells by Enhancing TGF-β1 Signaling
Source: PLoS One. 2013 Nov 18;8(11):e79857. doi: 10.1371/journal.pone.0079857 (PMC3832483; doi:10.1371/journal.pone.0079857)

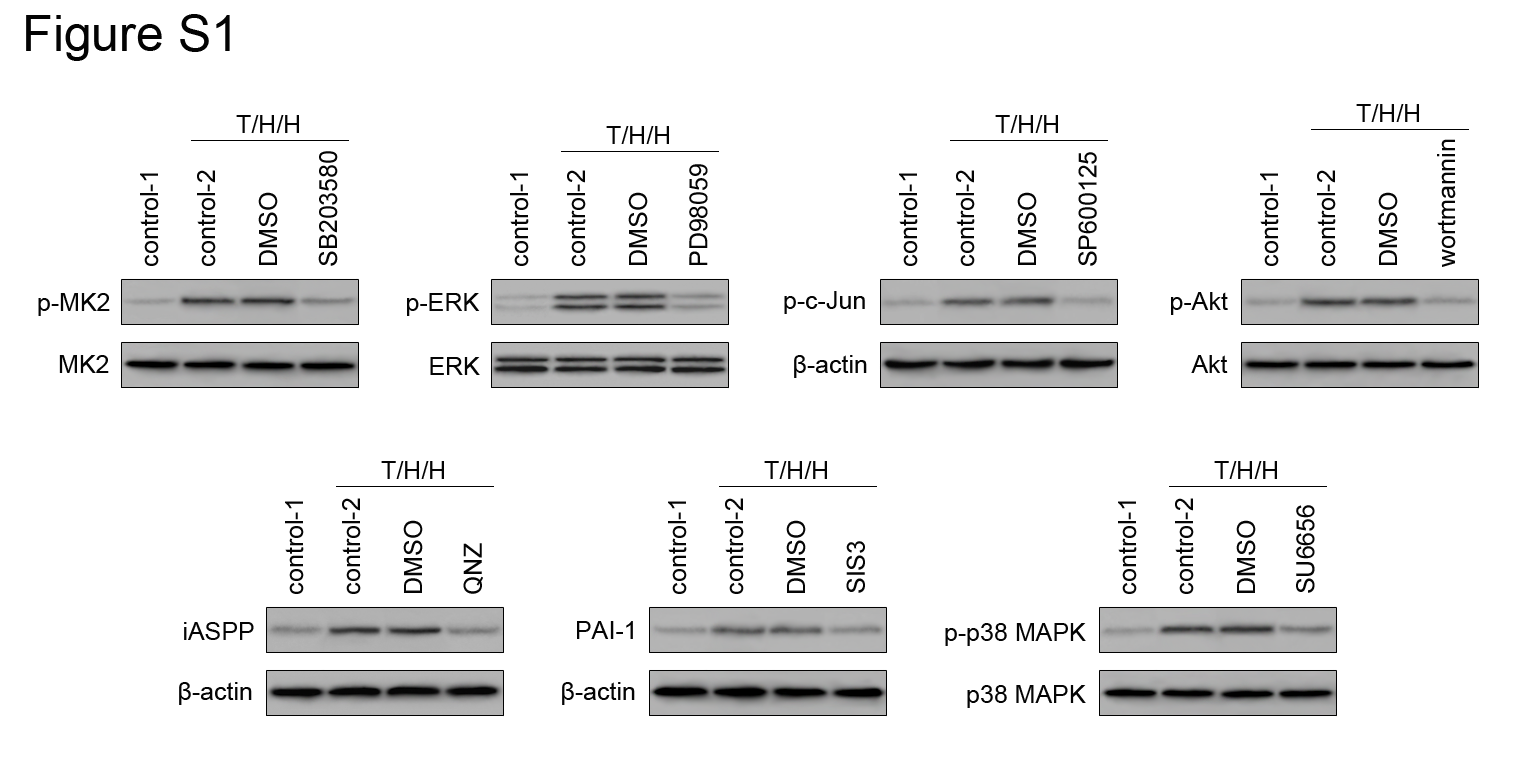

Supplement: Figure S1 — The inhibitory effect of inhibitors on signaling pathways. HepG2 cells were untreated or treated for 7 days with T/H/H in absence or presence of SB203580 (10 µM), PD98059 (10 µM), SP600125 (10 µM), wortmannin (WT, 40 nM), QNZ (40 nM), SIS3 (2 µM), and SU6656 (10 µM). The phosphorylation of MK2 was detected to demonstrate the inhibition of p38 MAPK by SB203580. The phosphorylation of ERK was detected to demonstrate the inhibition of MEK by PD98059. The phosphorylation of c-Jun was detected to demonstrate the inhibition of JNK by SP600125. The phosphorylation of Akt was detected to demonstrate the inhibition of PI3K by wortmannin. The expression of iASPP was detected to demonstrate the inhibition of NF-κB by QNZ. The expression of PAI-1 was detected to demonstrate the inhibition of Smad3 by SIS3. The phosphorylation of p38 MAPK was detected to demonstrate the inhibition of Src by SU6566. (TIF) [file pone.0079857.s001.tif]

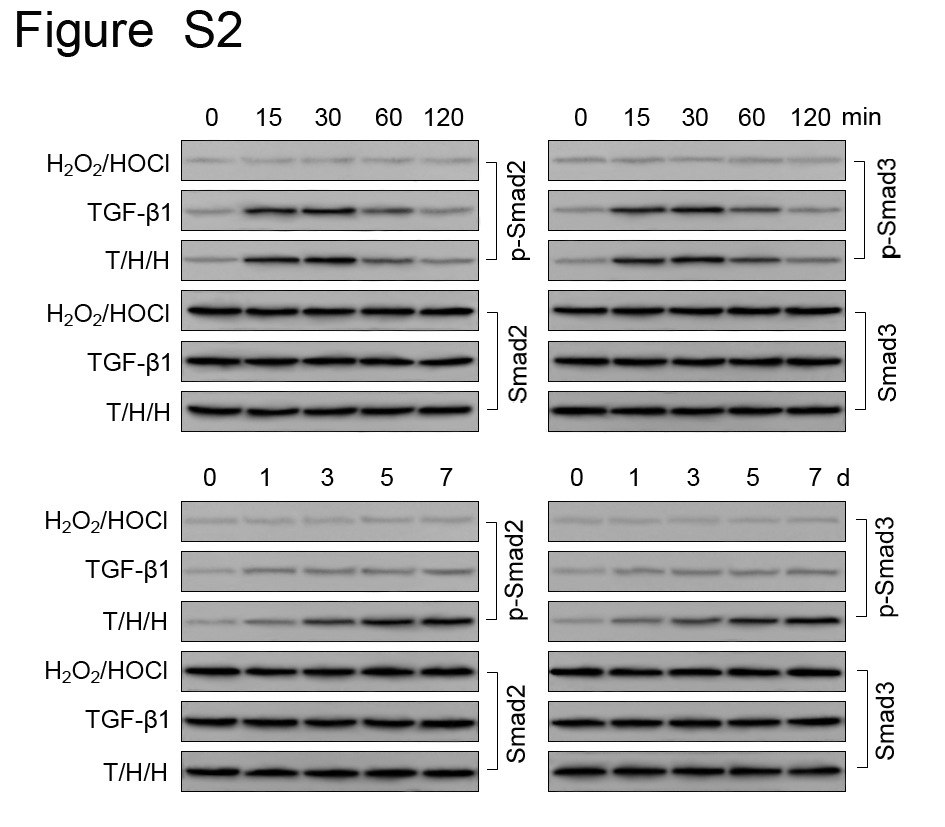

Supplement: Figure S2 — H2O2/HOCl promotes TGF-β1-induced sustained activation of Smad pathway. HepG2 cells were stimulated with H2O2/HOCl, TGF-β1, and T/H/H (TGF-β1, 5 ng/ml, H2O­2, 100 µM, HOCl, 50 µM). The phosphorylation of Smad2 and Smad3 was detected by Western blot at the indicated time points. (TIF) [file pone.0079857.s002.tif]

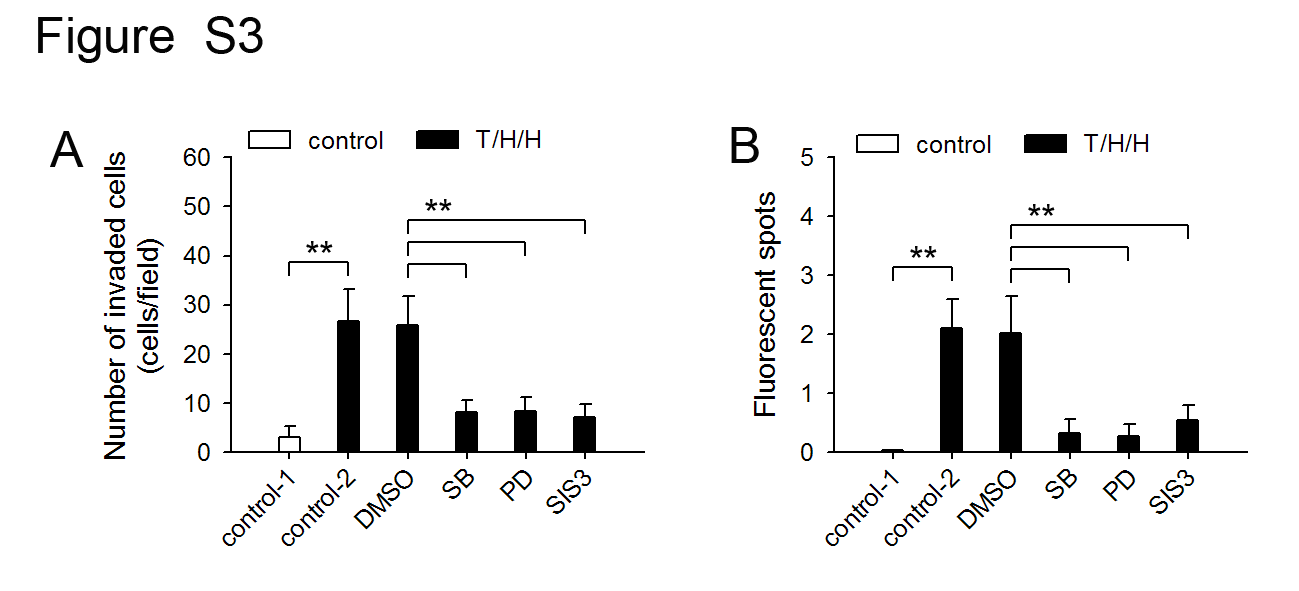

Supplement: Figure S3 — Sustained activation of signaling pathways is required for TGF-β1/H2O2/HOCl to promote invasion. HepG2 cells were cultured in absence or presence of T/H/H (TGF-β1/H2O2/HOCl). After 96-h culture, SB203580 (20 µM), PD98059 (20 µM), or SIS3 (2 µM) was added to the culture containing TGF-β1/H2O­2/HOCl. The cells were continuously cultured for another 6 days, and then used for the assay of invasive migration (A) and extravasation (B) as described in Methods. P values, *P<0.05, **P<0.01. (TIF) [file pone.0079857.s003.tif]

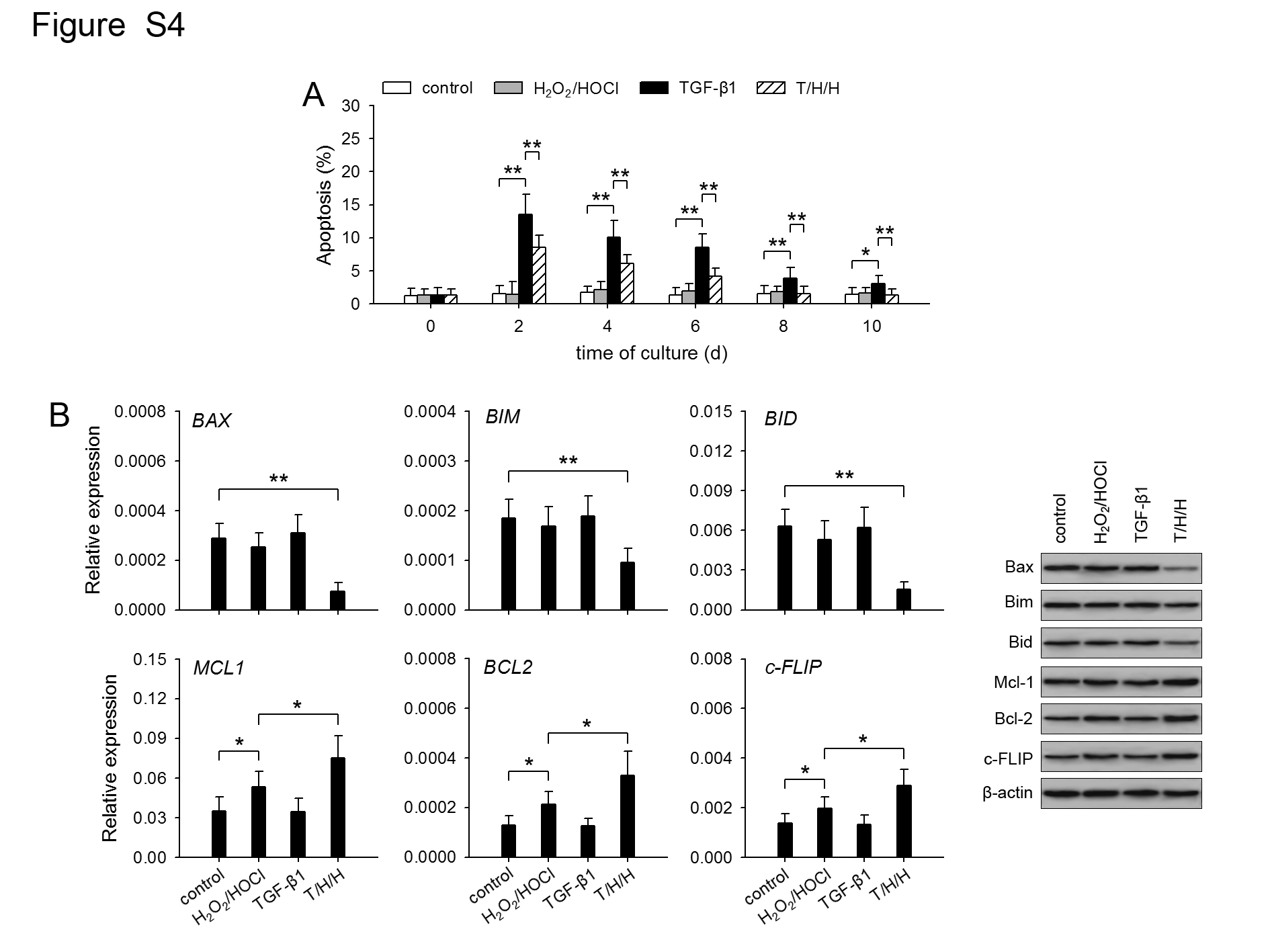

Supplement: Figure S4 — The effect of TGF-β1/H2O2/HOCl on apoptosis of HCC cells. (A) HepG2 cells were cultured in absence or presence of H2O2/HOCl, TGF-β1, and T/H/H (TGF-β1/H2O­2/HOCl). The apoptosis of the cells was detected at the indicated time points as described in Methods. (B) HepG2 cells were cultured for 10 days in absence or presence of H2O2/HOCl, TGF-β1, or T/H/H. The expression of BAX, BIM, BID, MCL1, BCL2, and c-FLIP was detected by real-time RT-PCR and Western blot. P values, *P<0.05, **P<0.01. (TIF) [file pone.0079857.s004.tif]

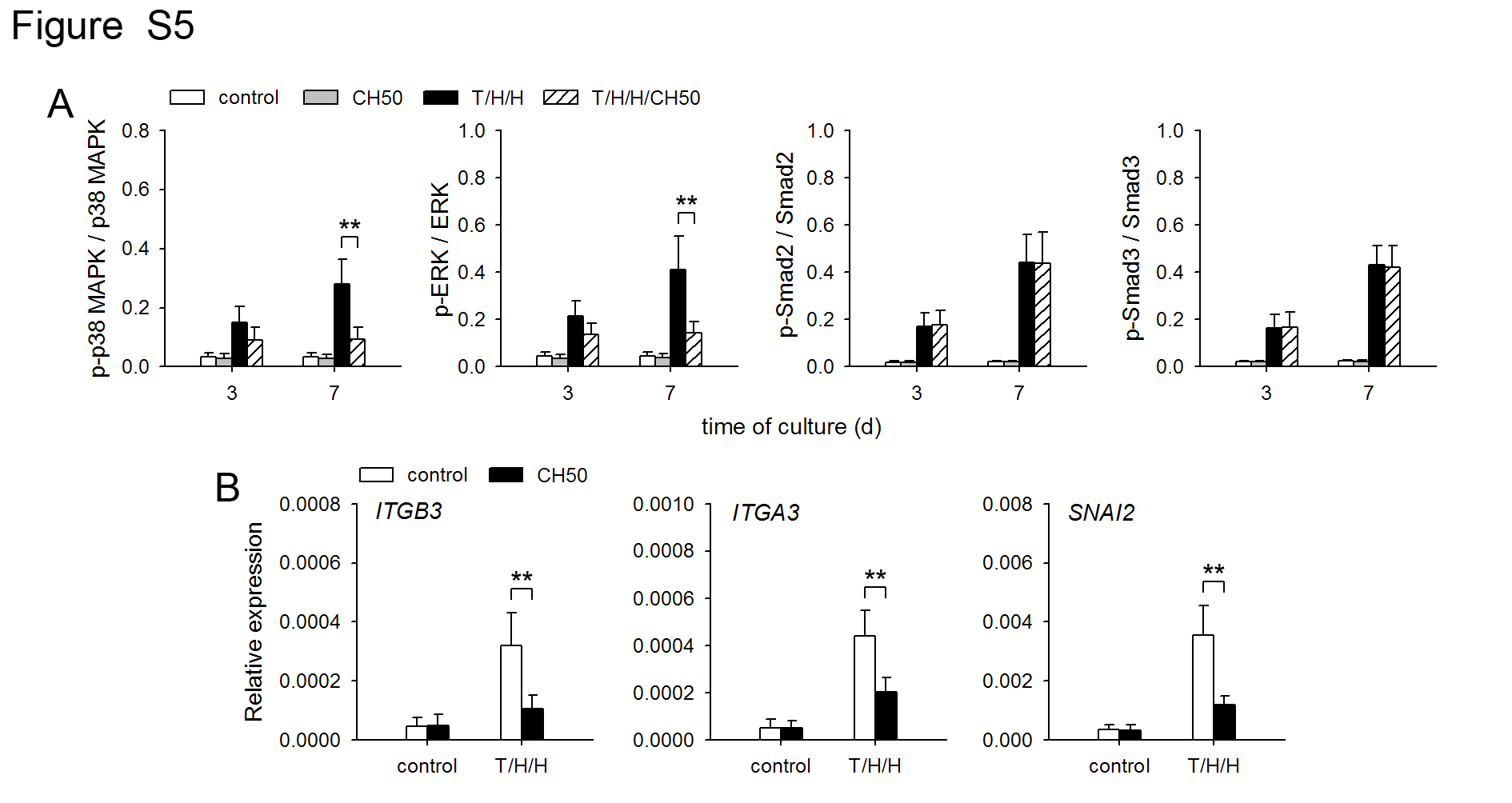

Supplement: Figure S5 — CH50 alters the effect of TGF-β1/H2O2/HOCl on HCC cells. (A) HepG2 cells were untreated or treated for the indicated time with T/H/H (TGF-β1/H2O2/HOCl) in absence or presence of CH50 (20 µg/ml). The relative activation of p38 MAPK (p-p38 MAPK/p38 MAPK), ERK (p-ERK/ERK), Smad2 (p-Smad2/Smad2), and Smad3 (p-Smad3/Smad3) was calculated after densitometric analysis of Western blots. (B) HepG2 cells were untreated or treated for 8 days with T/H/H in absence or presence of CH50. The expression of ITGB3, ITGA3, and SNAI2 genes was detected by real-time RT-PCR. P values, **P<0.01. (TIF) [file pone.0079857.s005.tif]
